# Gel-immersion endoscopy revealing hemobilia masquerading as duodenal stent-related bleeding

**DOI:** 10.1055/a-2946-2279

**Published:** 2026-09-15

**Authors:** Kohei Nishizawa, Junichi Kaneko, Haruna Takahashi, Daijiro Suzuki, Masaki Takinami, Yurimi Takahashi, Satoshi Tamura

**Affiliations:** 1 Department of Gastroenterology 13773 Iwata City HospitalIwataShizuoka PrefectureJapan; 2 Department of Hepatology 13773 Iwata City HospitalIwataShizuoka PrefectureJapan

Hemobilia is a rare but potentially life-threatening cause of gastrointestinal bleeding in patients with pancreatobiliary cancer. 1​ ​2​ In patients with an indwelling duodenal metallic stent (MS), diagnosis and management are challenging because the stent may obscure the major duodenal papilla, making it difficult to distinguish hemobilia from duodenal stent-related bleeding. 3​

Gel-immersion endoscopy (GIE) improves visualization during gastrointestinal bleeding by preventing rapid blood diffusion using transparent gels. 4​ ​5​ We reported a case in which GIE identified hemobilia mimicking duodenal stent-related bleeding, and fully covered self-expandable MS placement achieved hemostasis ( Video 1 ).

**Video 1** We report a case in which gel-immersion endoscopy identified hemobilia mimicking duodenal stent-related bleeding, and fully covered self-expandable metallic stent placement achieved hemostasis. Video URI:


A 55-year-old woman with pancreatic head cancer presented with hematemesis. Five months prior to presentation, she underwent biliary and duodenal MS deployment for pancreatic head cancer, followed by chemotherapy. Contrast-enhanced computed tomography revealed the migration of the biliary MS into the stomach, with no pseudoaneurysm or active extravasation. Upper endoscopy revealed no obvious active bleeding, and the migrated biliary MS was removed. Thereafter, no recurrent bleeding or biliary obstruction was observed; however, on hospital day 8, she developed recurrent hematemesis and underwent a repeat emergency upper endoscopy.

**Fig. 1:**
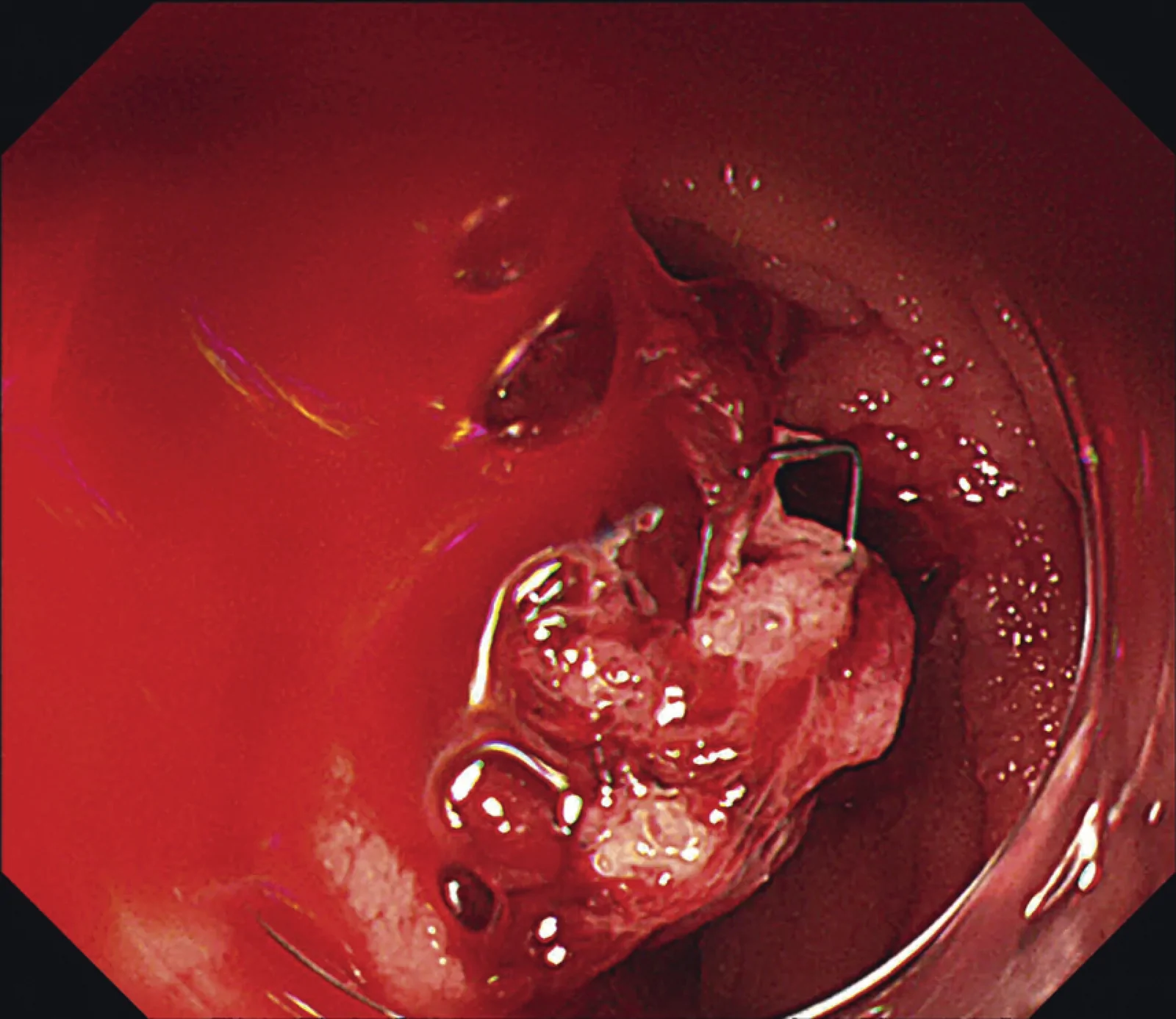
Oozing was observed beside the duodenal metallic stent.

**Fig. 2:**
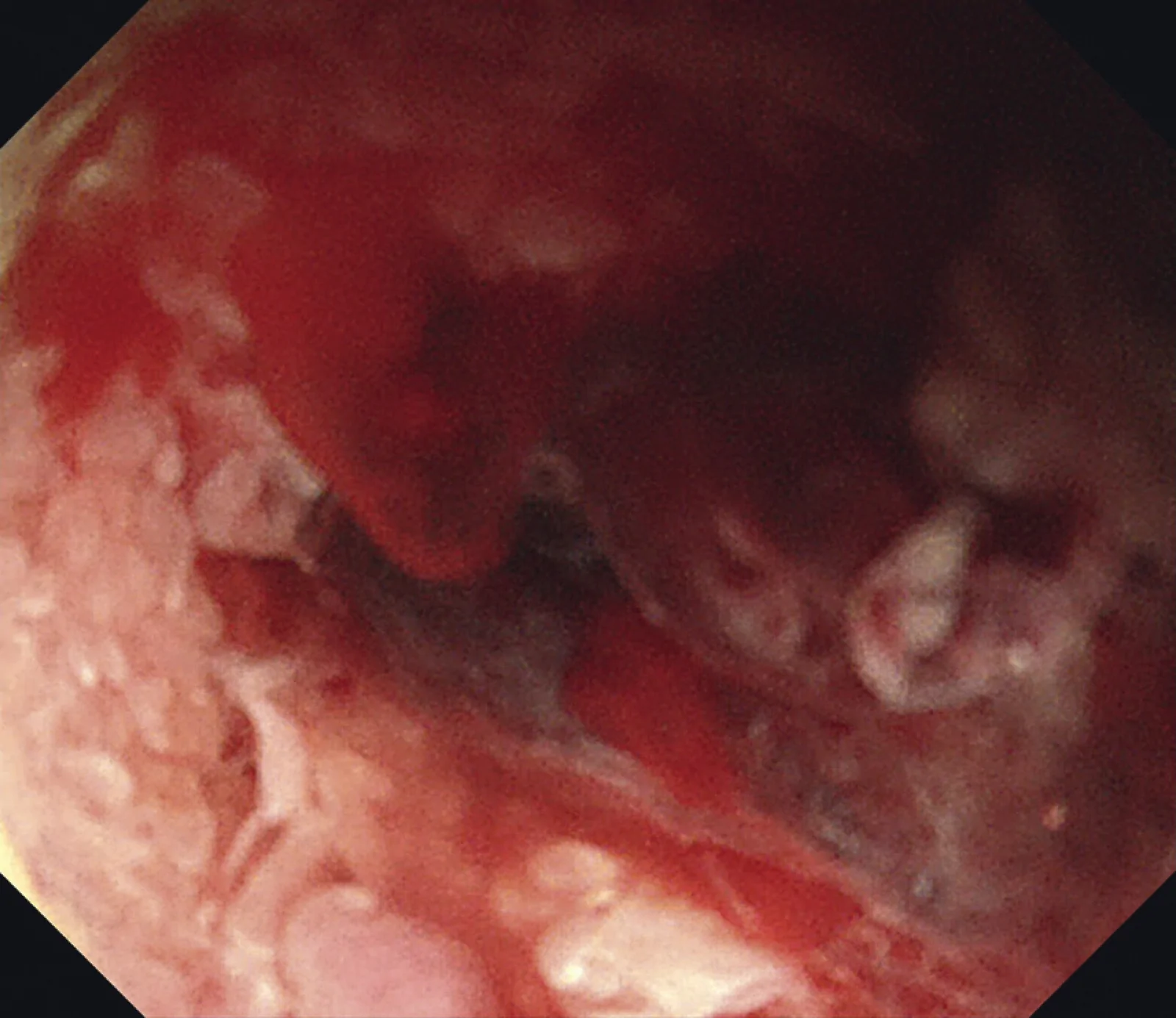
After gel immersion, visual field improved, and active bleeding from a small recess beside the duodenal metallic stent was clearly identified.

**Fig. 3:**
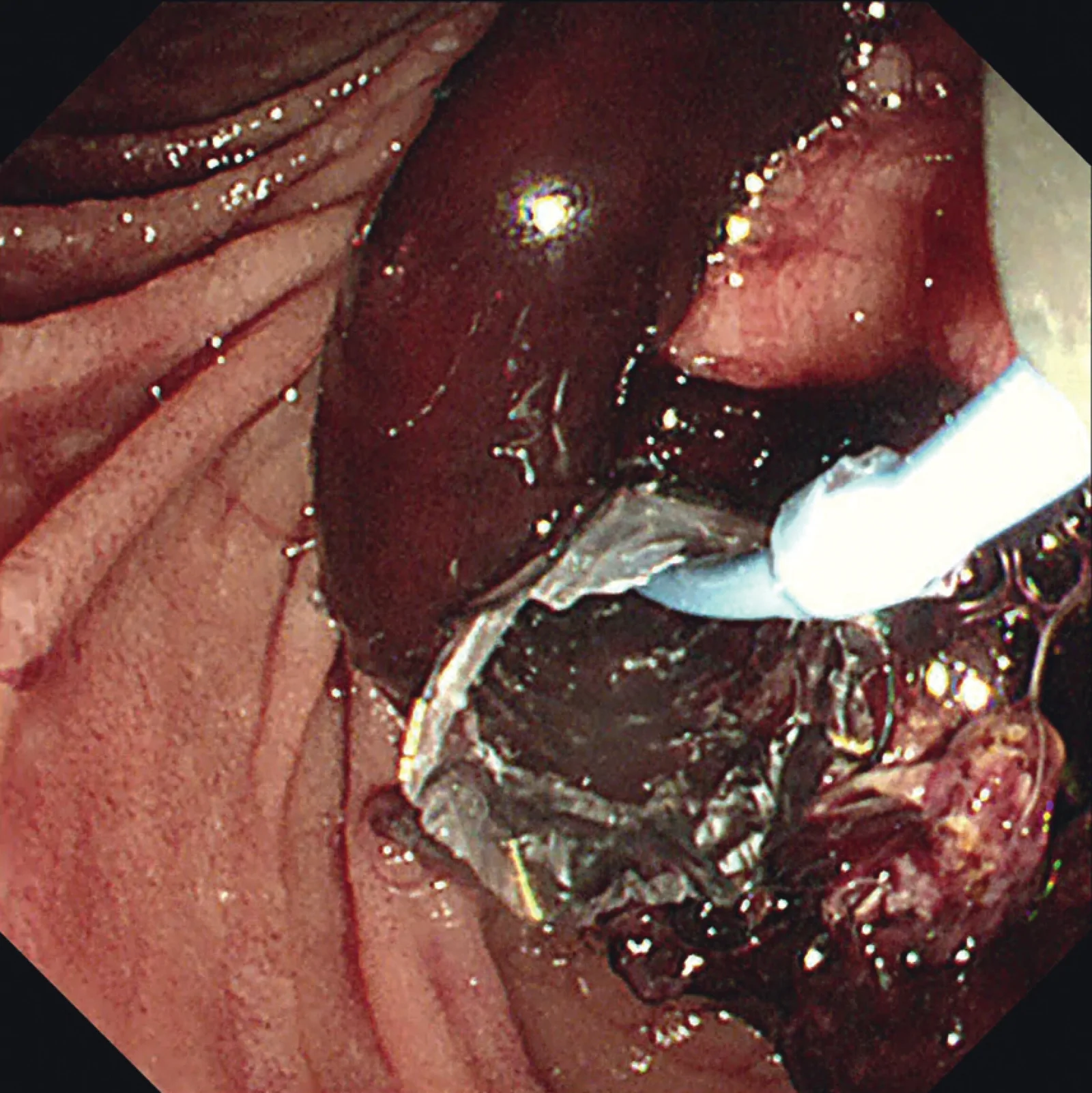
A fully covered self-expandable metallic stent was placed for tamponade hemostasis, successfully achieving hemostasis.

Forward-viewing endoscopy revealed oozing beside the duodenal MS; however, active bleeding and poor visibility prevented the precise localization of the bleeding point ( Fig. 1 ). VISCOCLEAR (Otsuka Pharmaceutical Factory, Tokushima, Japan) was injected into the duodenal lumen. After gel immersion, the visual field improved and active bleeding from a small recess beside the duodenal stent was clearly identified ( Fig. 2 ). A catheter and a guidewire were inserted into the small recess. Fluoroscopy confirmed access to the bile duct, which was filled with blood clots, thereby establishing the diagnosis of hemobilia. After switching to a duodenoscope, a fully covered self-expandable metallic stent was deployed in the bile duct, achieving immediate hemostasis ( Fig. 3 ). No recurrent bleeding occurred, and chemotherapy was resumed 3 weeks later.

Endoscopy_UCTN_Code_TTT_1AO_2AD
